# Purification of Ustiloxins A and B from Rice False Smut Balls by Macroporous Resins

**DOI:** 10.3390/molecules18078181

**Published:** 2013-07-11

**Authors:** Tijiang Shan, Weibo Sun, Xiaohan Wang, Xiaoxiang Fu, Wenxian Sun, Ligang Zhou

**Affiliations:** MOA Key Laboratory of Plant Pathology, Department of Plant Pathology, College of Agronomy and Biotechnology, China Agricultural University, Beijing 100193, China

**Keywords:** ustiloxins A and B, rice false smut balls, *Villosiclava virens*, macroporous resin, adsorption and desorption, purification

## Abstract

Ustiloxins are cyclopeptide mycotoxins produced by *Villosiclava virens*, the pathogenic fungus of rice false smut disease. Both resins SP207 and SP700 were screened to show the best adsorption and desorption properties for ustiloxins A and B among 20 commercial macroporous resins. Dynamic adsorption and desorption tests were carried out to optimize the process parameters. The optimal conditions for adsorption of resin SP207 were a processing volume as 32 bed volumes (BV), pH value of 4, and flow rate of 2 BV/h; and those for desorption of resin SP207 were a 40:60 (v/v) ratio of ethanol to water, an eluent volume of 4 BV, pH value of 4 and a flow rate of 3 BV/h. The optimal conditions for adsorption of resin SP700 were a processing volume of 26 BV, pH value as 4, flow rate of 2 BV/h; and those for desorption of resin SP700 were a 30:70 (v/v) ratio of ethanol to water solution as eluent, volume of 4 BV, pH value as 4 and flow rate of 2 BV/h. Under the optimal conditions; the purities of ustiloxins A and B obtained with resin SP207 increased 23.06-fold and 19.78-fold, respectively; and their recoveries were 96.67% and 81.25%; respectively. Similarly; the purities of ustiloxins A and B obtained with resin SP700 increased 14.75-fold and 15.33-fold and their recoveries were 93.65% and 88.64%; respectively. The results show that adsorption and desorption on SP207 and SP700 resins are effective strategies for purifying ustiloxins A and B. The developed methods are beneficial for large-scale preparation and purification of ustiloxins A and B from rice false smut balls.

## 1. Introduction

Ustiloxins are 13-membered cyclopeptides isolated from the water extract of the rice false smut disease pathogen *Villosiclava virens* Tanaka & Tanaka (anamorph: *Ustilaginoidea virens* Takahashi) [1,2,3]. They are toxic to plants and animals, especially due to their antimitotic activity, by inhibiting microtubule assembly and cell skeleton formation [4]. Of five elucidated ustiloxins, ustiloxins A and B are the predominant toxin components in rice false smut balls which were composed of the chlamydospores and mycelia of the pathogen [5]. Ustiloxins A and B have been proved to be more effective cytotoxins than other ustiloxins (*i.e.*, ustiloxins C, D and F) [6]. Ustiloxins A and B have been regarded as a novel resource with potential applications for medicinal and agrochemical purposes. They may act as anticancer agents for clinical application, and act as insecticides for agricultural production [7,8,9].

In order to speed up the investigation and application of ustiloxins, one of the most important approaches is to efficiently obtain ustiloxins A and B though it is very difficult to separate and purify ustiloxins A and B from other polar compounds such as carbohydrates and amino acids present in the water extract of rice false smut balls. As ustiloxins A and B have not been successfully synthesized up to now, rice false smut balls have been regarded as the only source of ustiloxins A and B [3].

Macroporous resins have been widely used in separation and purification of natural compounds from plants and microorganisms [10,11,12,13]. To the best of our knowledge, there was no information available about simultaneous enrichment and purification of ustiloxins A and B from rice false smut balls by using macroporous resins. The purpose of this investigation was to develop an efficient method for the preparative separation and purification of ustiloxins A and B from rice false smut balls. The adsorption and desorption properties of ustiloxins A and B on the macroporous resins with various polarities were studied in detail. The process parameters of two selected resins SP207 and SP700 were further optimized to purify ustiloxins A and B from rice false smut balls.

## 2. Results and Discussion

### 2.1. Adsorption and Desorption Propetries of the Resins

Adsorption and desorption properties of the resins for ustiloxins A and B are shown in Table 1. The non-polar macroporous resins exhibited better adsorption and desorption properties for ustiloxins A and B than the weakly-polar and polar resins. For ustiloxin A, SP207 possessed better adsorption capacity (1280.47 µg/g) and adsorption ratio (92.76%) than any other resin, followed by resins SP850, SP825L, SP700 and SP70, with adsorption ratios all above 80%. Resin SP700 showed the best desorption ratio (77.79%) for ustiloxin A among all tested resins (Table 1).

For ustiloxin B, the adsorption capacity of resin SP207 was higher than that of any other resin, followed by SP700 and SP850. Meanwhile, SP207 showed the highest desorption ratio (66.56%) for ustiloxin B, followed by SP700 with a desorption ratio of 60.28%.

Normally, the surface area and pore radius of the adsorbent maintain a linear relation with adsorption capacity for resins [14,15]. Among three aromatic resins (*i.e*., SP700, SP850 and SP825L), SP700 with the largest surface area (1 200 m^2^/g) presented the best adsorption and desorption capacity for ustiloxin B. SP207 is a highly porous styrene resin containing bromine chemically bonded to the styrene polymer matrix. Compared with the other adsorbents made of pure styrene–divinylbenzene, SP207 has a higher hydrophobicity which makes it be more efficient for adsorbing ustiloxins A and B. Thus, compared with other resins, SP207 and SP700 displayed better adsorption and desorption capacities for ustiloxins A and B, so they were selected for further study in the following adsorption kinetics experiments.

**Table 1:** Adsorption capacities, adsorption ratio and desorption ratio of the macroporous resins for ustiloxins A and B.

Resin	Ustiloxin A			Ustiloxin B		
	Adsorption capacity (µg/g)	Adsorption ratio (%)	Desorption ratio (%)	Adsorption capacity (µg/g)	Adsorption ratio (%)	Desorption ratio (%)
X-5	957.05 ± 6.23 ^i^	69.32 ± 0.51 ^i^	61.26 ± 3.19 ^e^	583.32 ± 5.74 ^i^	53.70 ± 0.57 ^i^	44.84 ± 0.40 ^f^
D-101	1059.02 ± 1.06 ^f^	76.80 ± 0.21 ^f^	68.90 ± 2.62 ^cd^	710.78 ± 2.30 ^f^	65.51 ± 0.25 ^f^	48.34 ± 3.37 ^e^
HPD-100	984.66 ± 7.35 ^g^	71.29 ± 0.49 ^g^	67.27 ± 1.01 ^d^	696.50 ± 3.78 ^g^	64.09 ± 0.38 ^g^	33.12 ± 1.43 ^g^
D1300	974.44 ± 21.06 ^gh^	70.64 ± 1.50 ^gh^	55.86 ± 0.35 ^f^	698.83 ± 6.30 ^g^	64.39 ± 0.60 ^g^	34.06 ± 0.74 ^g^
D3520	984.11 ± 2.35 ^g^	71.31 ± 0.22 ^g^	60.02 ± 1.51 ^e^	692.43 ± 4.80 ^g^	63.77 ± 0.41 ^g^	33.36 ± 2.13 ^g^
AB-8	966.90 ± 2.81 ^hi^	70.09 ± 0.31 ^hi^	61.10 ± 2.96 ^e^	553.74 ± 14.54 ^j^	51.02 ± 1.42 ^j^	55.32 ± 2.72 ^cd^
DM130	925.70 ± 9.60 ^j^	67.05 ± 0.70 ^j^	60.73 ± 1.09 ^e^	660.77 ± 0.38 ^h^	60.83 ± 0.07 ^h^	33.15 ± 3.18 ^g^
DS-8	480.66 ± 12.24 ^l^	34.80 ± 0.88 ^l^	41.76 ± 0.99 ^g^	559.93 ± 2.69 ^j^	51.53 ± 0.26 ^j^	9.76 ± 1.07 ^i^
DM-301	376.93 ± 4.76 ^m^	27.30 ± 0.35 ^m^	20.65 ± 2.59 ^i^	519.25 ± 1.89 ^k^	47.79 ± 0.13 ^k^	5.62 ± 1.19 ^j^
ADS-17	92.02 ± 2.82 ^p^	6.66 ± 0.20 ^p^	7.53 ± 2.16 ^kj^	58.14 ± 5.96 ^o^	5.35 ± 0.54 ^o^	14.59 ± 1.40 ^h^
S-8	905.34 ± 8.66 ^k^	65.56 ± 0.54 ^k^	6.87 ± 1.09 ^k^	668.30 ± 14.22 ^h^	61.50 ± 1.22 ^h^	8.85 ± 1.12 ^i^
NKA-9	166.16 ± 1.81 ^o^	12.04 ± 0.12 ^o^	10.72 ± 0.93 ^j^	235.61 ± 2.34 ^n^	21.70 ± 0.20 ^n^	2.30 ± 0.14 ^k^
DA-201	220.49 ± 7.40 ^n^	15.96 ± 0.54 ^n^	31.21 ± 1.55 ^h^	464.81 ± 3.14 ^l^	42.77 ± 0.32 ^l^	7.06 ± 0.72 ^ij^
HP-20	1062.66 ± 5.82 ^f^	76.97 ± 0.45 ^f^	73.29 ± 3.82 ^b^	727.13 ± 18.56 ^e^	66.93 ± 1.66 ^e^	53.82 ± 1.71 ^d^
SP825L	1147.75 ± 5.21 ^c^	83.18 ± 0.35 ^c^	73.81 ± 0.20 ^b^	781.05 ± 2.95 ^c^	71.94 ± 0.29 ^c^	57.68 ± 1.40 ^bc^
SP850	1181.15 ± 6.19 ^b^	85.56 ± 0.37 ^b^	68.56 ± 0.92 ^cd^	817.18 ± 1.67 ^b^	75.23 ± 0.06 ^b^	56.72 ± 1.78 ^cd^
SP700	1129.91 ± 4.40 ^d^	81.87 ± 0.22 ^d^	77.79 ± 0.35 ^a^	821.12 ± 2.86 ^b^	75.62 ± 0.27 ^b^	60.28 ± 1.19 ^b^
SP70	1104.00 ± 1.68 ^e^	80.02 ± 0.12 ^e^	71.19 ± 1.91 ^bc^	748.11 ± 1.89 ^d^	68.92 ± 0.14 ^d^	55.19 ± 2.17 ^cd^
SP207	1280.47 ± 1.41 ^a^	92.76 ± 0.11 ^a^	73.64 ± 4.59 ^b^	946.80 ± 7.07 ^a^	87.17 ± 0.70 ^a^	66.56 ± 0.57 ^a^
HP_2_MGL	64.14 ± 8.95 ^q^	4.65 ± 0.65 ^q^	21.18 ± 2.78 ^i^	414.32 ± 0.65 ^m^	38.15 ± 0.08 ^m^	1.27 ± 0.21 ^k^

Note: The values are expressed as means ± standard deviations (*n* = 3). Different letters indicate significant differences among the treatments in each column at *p* = 0.05 level.

### 2.2. Static Adsorption Kinetics of Resins SP207 and SP700 for Ustiloxins A and B

**Figure 1:**
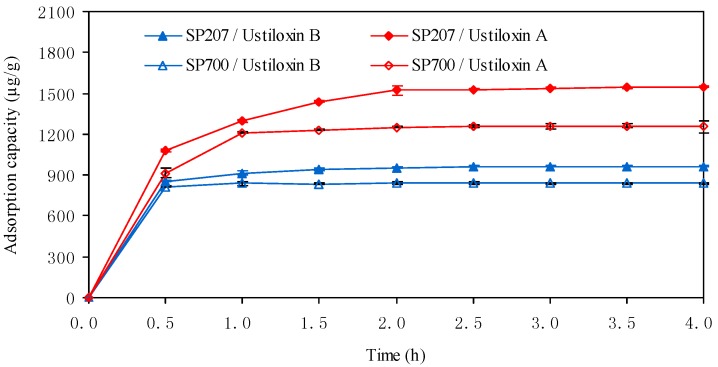
Static adsorption kinetics curves of resins SP207 and SP700 for ustiloxins A and B.

Though adsorption and desorption capacities of resins are important indicators for the evaluation of macroporous resins, a more suitable resin must have a higher adsorption ratio [16]. The static adsorption kinetics curves of resins SP207 and SP700 for ustiloxins A and B are shown in Figure 1. The adsorption capacity for ustiloxin A reached equilibrium at either 2.0 h on SP207 or 1.0 h on SP700. On the other hand, the adsorption capacity for ustiloxin B reached equilibrium either at 1.5 h on SP207 or 1.0 h on SP700. From the above results, SP207 had a bigger adsorption capacity for ustiloxins A and B than SP700, and SP700 had a faster adsorption ratio for ustiloxins A and B than SP207, so both two resins were selected for further investigation.

### 2.3. Adsorption Isotherms of Resins SP207 and SP700 for Ustiloxins A and B

**Figure 2:**
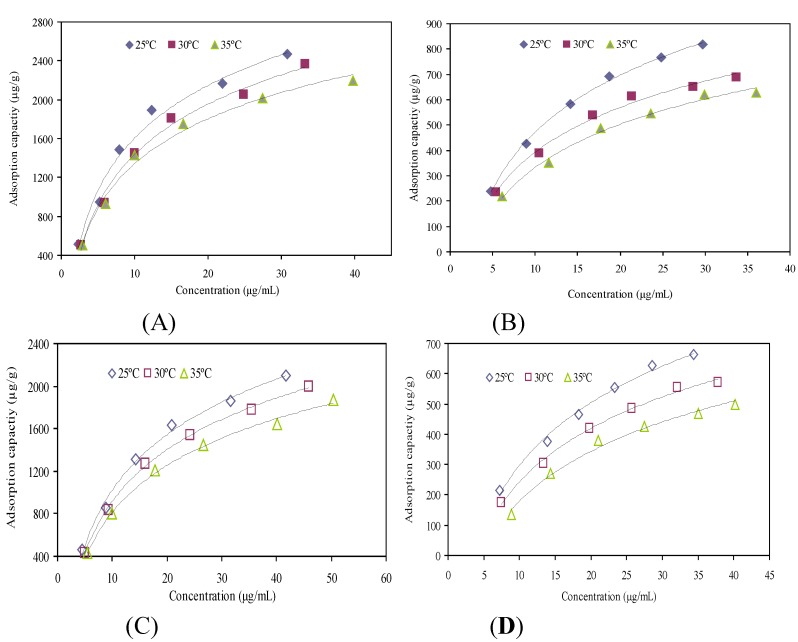
Adsorption isotherms of SP207 for ustiloxin A (**A**), SP207 for ustiloxin B (**B**), SP700 for ustiloxin A (**C**), and SP700 for ustiloxin B (**D**) at 25, 30 and 35 °C, respectively.

The adsorption isotherms of resins SP207 and SP700 for ustiloxins A and B were investigated with different concentrations of the sample solutions at 25 °C, 30 °C and 35 °C, respectively, which are depicted in Figure 2.

The initial concentrations of ustiloxin A were 19.89, 37.54, 58.68, 76.27, 95.24 and 113.44 µg/mL, and those of ustiloxin B were 13.47, 23.55, 33.97, 42.13, 50.77 and 57.06 µg/mL. As shown in Figure 2, the adsorption capacities of both SP207 and SP700 increased with the increase of the initial concentrations, and reached saturation when the initial concentrations of ustiloxins A and B were 113.44 and 57.06 µg/mL, respectively, and the concentration of crude extract was about 60 mg/mL.

Equilibrium data give information about the affinity between solute and adsorbent. Both the Langmuir isotherm model and Freundlich isotherm model are the most often used models for the adsorption of solutes from adsorbent [12]. They can indicate the interaction between solutes and resins, and show the linear relationship between them. Langmuir and Freundlich model-fitting results of the obtained data of two targeted ustiloxins are listed in Table 2. Comparing these two models, the linear correlative coefficients (*R*^2^) indicated that Langmuir equation fitted the experimental data better than Freundlich equation. The *R*^2^ values for the Langmuir equation were all above 0.97, but those for the Freundlich equation were between 0.92 and 0.99. Meanwhile, for the Freundlich equation, the adsorption can easily take place when 1/n value is between 0.1 and 0.5, however, this tends not to happen when the 1/n value is between 0.5 and 1.0, and is almost impossible to occur when the 1/n value exceeds 1.0 [16]. In ustiloxins A and B’s Frendlich equation, all 1/n values are between 0.5 and 1.0 (Table 2). Even though the *R*^2^ values of ustiloxins B’s Freundlich equation are above 0.96, it is hard for the adsorption on SP207 to happen. Thus, Langmuir equation can better describe the adsorption and desorption behaviors of ustiloxins A and B on resin SP207 than Freundlich equation. In addition, for the same initial concentration of sample solutions, the adsorption capacities of SP207 for ustiloxins A and B were bigger at low temperature than those at high temperature in the tested temperature range (Figure 2), which indicated that the adsorption process was exothermic. Meanwhile, *Q*_max_ also decreased with increasing temperature for two targeted ustloxins (Table 2).

Similarly, the Langmuir model was also more suitable to describe the adsorption and desorption behaviors of ustiloxins A and B on resin SP700 than the Freundlich model. From the results shown in Figure 2, at the same initial concentration, the adsorption capacities of resin SP700 decreased with increasing temperature in the investigated temperature range, which indicated that the adsorption was a thermopositive process. Meanwhile, *Q*_max_ also decreased with increasing temperature for all two targeted ustiloxins. For ustiloxin A, *Q*_max_ decreased from 4593.73 µg/g at 25 °C to 3628.93 µg/g at 35 °C; and for ustiloxin B, *Q*_max_ decreased from 1732.89 µg/g at 25 °C to 976.10 µg/g at 35 °C (Table 2). Therefore, 25 °C was selected in the following experiments.

**Table 2:** Langmuir and Freundlich parameters of ustiloxins A and B on resins SP207 and SP700.

Resin	Adsorbate	Temperature (°C)	Langmuir equation			Freundlich equation		
			*Q*_max_	*K*_L_	*R*^2^	*K*_F_	N	*R*^2^
SP207	Ustiloxin A	25	4189.20	245.97	0.9926	347.38	1.64	0.9410
		30	3682.28	218.12	0.9979	309.17	1.64	0.9637
		35	3482.60	205.48	0.9942	327.79	1.80	0.9358
	Ustiloxin B	25	1798.79	58.11	0.9944	90.01	1.48	0.9718
		30	1207.39	54.08	0.9964	92.19	1.68	0.9662
		35	1162.41	43.53	0.9969	71.65	1.58	0.9697
SP700	Ustiloxin A	25	4593.73	114.26	0.9947	184.93	1.47	0.9613
		30	4389.28	99.81	0.9879	171.99	1.50	0.9581
		35	3628.93	91.66	0.9875	168.19	1.57	0.9563
	Ustiloxin B	25	1732.89	34.09	0.9988	51.81	1.35	0.9878
		30	1667.51	26.47	0.9950	41.63	1.34	0.9780
		35	976.10	26.92	0.9789	25.92	1.20	0.9287

### 2.4. Effects of pH Values of Sample Solution on Adsorption Capacities of Resins SP207 and SP700

Considering that the pH value of the sample solution is a very important parameter for the performance of macroporous resins, the influences of initial pH values of the sample solution on the adsorption capacities of resins SP207 and SP700 for ustiloxins A and B were studied, and the results are shown in Table 3.

**Table 3:** Effects of pH values of sample solution on the adsorption capacities of resins SP207 and SP700.

pH value	Adsorption capacity of SP207 (µg/g)		Adsorption capacity of SP700 (µg/g)	
	Ustiloxin A	Ustiloxin B	Ustiloxin A	Ustiloxin B
3	1177.48 ± 1.94 ^c^	480.67 ± 3.08 ^c^	1190.74 ± 6.76 ^b^	475.38 ± 1.77 ^b^
4	1362.09 ± 12.57 ^a^	597.18 ± 11.54 ^a^	1335.30 ± 3.46 ^a^	547.49 ± 13.43 ^a^
5	1137.21 ± 1.81 ^d^	534.39 ± 3.49 ^b^	1070.76 ± 2.33 ^c^	427.96 ± 20.62 ^bc^
6	1221.80 ± 1.38 ^b^	511.89 ± 7.06 ^b^	1086.21 ± 29.40 ^c^	389.68 ± 10.62 ^cd^
7	1240.01 ± 36.26 ^b^	517.89 ± 2.13 ^b^	1106.75 ± 35.48 ^c^	349.63 ± 56.85 ^d^
8	1161.15 ± 3.60 ^cd^	303.10 ± 29.91 ^d^	1037.77 ± 45.67 ^c^	76.89 ± 16.50 ^e^
9	1021.51 ± 15.59 ^e^	93.87 ± 0.95 ^e^	945.07 ± 0.71 ^d^	56.04 ± 4.31 ^e^
10	718.44 ± 5.78 ^f^	101.88 ± 1.91 ^e^	538.59 ± 53.25 ^e^	58.43 ± 4.05 ^e^

Note: The values are expressed as means ± standard deviations (*n* = 3). Different letters indicate significant differences among the treatments in each column at *p* = 0.05 level.

Resins SP207 and SP700 exhibited the same tendency. When the initial pH values of sample solution were above 8, the adsorption capacities of resins rapidly decreased. For resin SP207, the adsorption capacities all reached maximum for ustiloxins A and B at pH 4, which were 1362.09 ± 12.57 µg/g and 597.18 ± 11.54 µg/g, respectively. On the other hand, for resin SP700, the maximum adsorption capacities for ustiloxins A and B were 1335.30 ± 3.46 µg/g and 547.49 ± 13.43 µg/g, respectively, at pH 4. Therefore, pH 4 was selected for initial pH of the sample solution in the following experiments.

### 2.5. Effects of pH Values of Ethanol Solution on Desorption Ratios and Purities of Ustiloxins

The effects of pH values of the ethanol solution on desorption ratios and purities of ustiloxins A and B for resins SP207 and SP700 were studied, and the results are shown in Table 4.

**Table 4:** Effects of pH values of ethanol solution on adsorption ratios and purities of ustiloxins A and B for resins SP207 and SP700.

Resin	pH value	Ustiloxin A		Ustiloxin B	
		Desorption ratio (%)	Purity (%)	Desorption ratio (%)	Purity (%)
SP207	3	29.26 ± 2.02 ^d^	1.14 ± 0.01 ^b^	43.78 ± 3.96 ^d^	0.77 ± 0.02 ^b^
	4	63.54 ± 3.27 ^a^	2.20 ± 0.18 ^a^	77.36 ± 3.88 ^a^	1.21 ± 0.11 ^a^
	5	58.60 ± 2.54 ^abc^	1.87 ± 0.02 ^a^	68.42 ± 0.15 ^abc^	0.99 ± 0.03 ^ab^
	6	51.67 ± 1.78 ^c^	2.35 ± 0.10 ^a^	65.72 ± 2.51 ^c^	1.34 ± 0.10 ^a^
	7	53.41 ± 3.01 ^bc^	2.13 ± 0.17 ^a^	63.91 ± 3.46 ^bc^	1.14 ± 0.16 ^a^
	8	55.97 ± 2.43 ^bc^	2.19 ± 0.23 ^a^	68.57 ± 1.44 ^bc^	1.20 ± 0.15 ^a^
	9	60.49 ± 3.03 ^ab^	2.22 ± 0.15 ^a^	70.63 ± 1.18 ^ab^	1.19 ± 0.09 ^a^
	10	55.63 ± 2.59 ^bc^	2.13 ± 0.09 ^a^	68.57 ± 1.92 ^bc^	1.18 ± 0.03 ^a^
	11	57.45 ± 4.62 ^bc^	2.29 ± 0.18 ^a^	70.05 ± 1.14 ^bc^	1.23 ± 0.18 ^a^
SP700	3	45.11 ± 0.50 ^c^	1.22 ± 0.11 ^b^	53.61 ± 2.95 ^d^	0.59 ± 0.05 ^b^
	4	74.05 ± 2.21 ^a^	2.25 ± 0.05 ^a^	77.57 ± 4.08 ^a^	0.94 ± 0.01 ^a^
	5	72.57 ± 0.32 ^ab^	2.34 ± 0.12 ^a^	73.55 ± 1.10 ^ab^	0.97 ± 0.14 ^a^
	6	70.76 ± 0.21 ^ab^	2.69 ± 0.05 ^a^	67.66 ± 1.15 ^c^	1.10 ± 0.01 ^a^
	7	70.22 ± 0.47 ^ab^	2.36 ± 0.04 ^a^	68.31 ± 2.30 ^bc^	0.96 ± 0.02 ^a^
	8	72.33 ± 4.35 ^ab^	2.52 ± 0.05 ^a^	69.73 ± 2.10 ^bc^	1.01 ± 0.04 ^a^
	9	66.71 ± 2.88 ^b^	2.36 ± 0.13 ^a^	65.36 ± 2.44 ^bc^	0.98 ± 0.12 ^a^
	10	72.15 ± 0.34 ^ab^	2.39 ± 0.12 ^a^	70.23 ± 0.49 ^bc^	0.98 ± 0.04 ^a^
	11	71.40 ± 3.07 ^ab^	2.32 ± 0.07 ^a^	69.80 ± 3.37 ^bc^	0.96 ± 0.04 ^a^

Note: The values are expressed as means ± standard deviations (*n* = 3). Different letters indicate significant differences among the treatments in each column including different resins and their pH values at *p* = 0.05 level.

The highest desorption ratio (63.54 ± 3.27%) was observed at pH 4 for ustiloxin A on resin SP207. Desorption ratios slightly decreased with increase of pH values. The purity (2.35 ± 0.10%) of ustiloxin A was the highest at pH 6. There were no significant differences among the purities of ustiloxin A when pH values of ethanol solution were between 4 and 11. Similarly, the highest desorption ratio (77.36 ± 3.88%) was observed at pH 4 for ustiloxin B on resin SP207. Therefore, pH value as 4 was determined as the optimum pH value of the ethanol solution for ustiloxins A and B desorption on resin SP207. Very similar results were observed about the effects of pH values of ethanol solution on desorption ratio and purities of ustiloxins A and B for resin SP700 (Table 4). Therefore, pH 4 ethanol solution was selected for resins SP207 and SP700 in the following experiments based on the desorption ratios and purities of ustiloxins A and B. 

### 2.6. Effects of Ethanol Concentration on Desorption Ratios and Purities of Ustiloxins A and B

Effects of ethanol concentration on desorption ratios and purities of ustiloxins A and B for resins SP207 and SP700 were investigated, and the results are shown in Table 5. For resin SP207, the desorption ratios of ustiloxins A and B increased rapidly with an increase in ethanol concentration and reached their peak values (74.68 ± 2.03% for ustiloxin A, and 85.76 ± 4.07% for ustiloxin B) when the ethanol concentration was at 40%. No significant differences were observed among the purities of ustiloxins A and B with different concentrations of ethanol solutions, so 40% ethanol solution was selected for resin SP207 as the appropriate desorption solution and used in the successive dynamic desorption experiments. Similarly, for resin SP700, 30% ethanol solution was selected in the successive dynamic desorption experiments.

**Table 5:** Effects of ethanol concentration on desorption ratios of resins SP207 and SP700 along with the purities of ustiloxins A and B.

Resin	Ethanol concentration (%, v/v)	Ustiloxin A		Ustiloxin B	
		Desorption ratio (%)	Purity (%)	Desorption ratio (%)	Purity (%)
SP207	10	34.06 ± 2.05 ^d^	1.60 ± 0.43 ^b^	61.11 ± 2.74 ^b^	1.08 ± 0.27 ^a^
	20	58.04 ± 3.82 ^c^	2.22 ± 0.14 ^ab^	77.43 ± 3.50 ^a^	1.12 ± 0.10 ^a^
	30	64.95 ± 4.80 ^bc^	2.33 ± 0.42 ^ab^	80.66 ± 5.44 ^a^	1.09 ± 0.21 ^a^
	40	74.68 ± 2.03 ^a^	2.42 ± 0.41 ^a^	85.76 ± 4.07 ^a^	1.03 ± 0.16 ^a^
	50	68.96 ± 3.45 ^ab^	2.05 ± 0.22 ^ab^	80.88 ± 4.62 ^a^	0.89 ± 0.10 ^a^
	70	68.06 ± 4.12 ^ab^	2.30 ± 0.23 ^ab^	79.46 ± 5.17 ^a^	0.98 ± 0.09 ^a^
SP700	10	41.39 ± 0.39 ^d^	2.47 ± 0.44 ^a^	67.58 ± 5.65 ^a^	1.30 ± 0.37 ^a^
	20	60.63 ± 4.00 ^c^	3.24 ± 0.42 ^a^	73.85 ± 2.83 ^a^	1.31 ± 0.51 ^a^
	30	69.09 ± 2.78 ^ab^	3.66 ± 0.43 ^a^	72.72 ± 3.65 ^a^	1.41 ± 0.53 ^a^
	40	70.14 ± 2.73 ^a^	3.25 ± 0.32 ^a^	72.91 ± 1.70 ^a^	1.23 ± 0.30 ^a^
	50	71.47 ± 0.81 ^a^	2.82 ± 0.40 ^a^	74.00 ± 1.14 ^a^	1.08 ± 0.24 ^a^
	70	65.50 ± 0.80 ^b^	2.64 ± 0.44 ^a^	67.83 ± 4.26 ^a^	1.03 ± 0.18 ^a^

Note: The values are expressed as means ± standard deviations (*n* = 3). Different letters indicate significant differences among the treatments in each column including different resins and their ethanol concentrations at *p* = 0.05 level.

### 2.7. Dynamic Adsorption and Desorption of Resins SP270 and SP700 for Ustiloxins A and B

#### 2.7.1. Dynamic Breakthrough Curves of Resins SP207 and SP700 for Ustiloxins A and B

The dynamic breakthrough curve is an important characteristic for the operation and dynamic response of an adsorption column. The breakthrough point is defined as the volume of loaded sample solution when the concentration of effluents accounts for one-tenth of that in the initial sample solution. When the adsorption capacity of resin decreased and even lost, the sample solution would have a leakage or waste, so we consider that the resin has been saturated when the concentration of effluents accounts for about one-tenth of that in the initial sample solution [12,17].

First of all, the study was performed in order to determine adsorption kinetics of ustiloxins A and B on resins SP207 and SP700, as well as to choose the flow rate to be used in the next optimization. The sample solution was prepared by diluting crude extract of rice false smut balls at a concentration of 60 mg/mL, the pH value was set at 4, and the initial concentrations of ustiloxins A and B in the sample solution were 107.39 µg/mL and 62.02 µg/mL, respectively. The curves were stopped until concentrations of ustiloxins A and B in effluents were equal to those in the sample solutions. 

**Figure 3:**
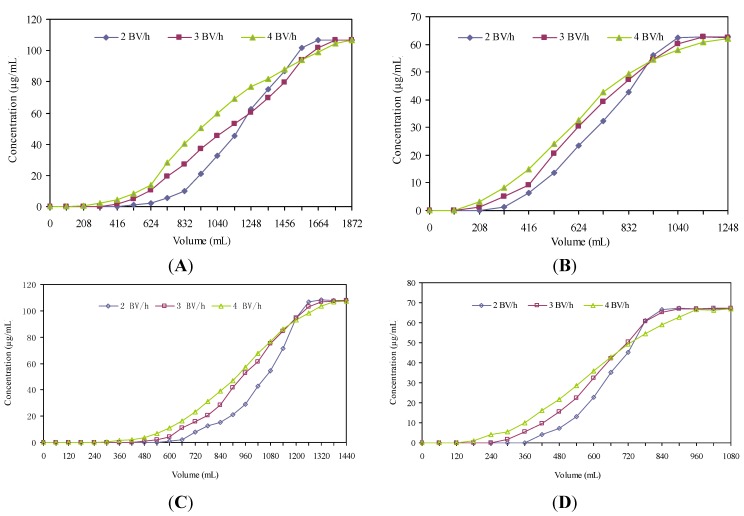
Dynamic breakthrough curves of ustiloxin A on the column packed with SP207 (**A**), ustiloxin B on the column packed with SP207 (**B**), ustiloxin A on the column packed with SP700 (**C**), and ustiloxin B on the column packed with SP700 (**D**).

As seen in Figure 3, both SP207 and SP700 displayed better adsorption properties for ustiloxin A or B at a flow rate of 2 bed volumes (BV)/h. Therefore, 2 BV/h was chosen as the most appropriate flow rate in subsequent experiments for SP207 and SP700.

For resin SP207, it was found that the breakthrough point of ustiloxin A [32 BV (832 mL)] was later than that of ustiloxin B, which was 16 BV (416 mL). To ensure both targeted ustiloxins reached their adsorption equilibrium, the sample solution was loaded at 32 BV in the following experiments. In addition, the adsorbtion capacities for ustiloxins A and B on resin SP207 were 87.38 mg and 25.02 mg, respectively, at their breakthrough points.

For resin SP700, the breakthrough point of ustiloxin A [26 BV (780 mL)] was also later than that of ustiloxin B, which was 16 BV (480 mL). To ensure both targeted ustiloxins reached their adsorption equilibrium, the sample solution was loaded at 26 BV in the following experiments. In addition, the adsorbtion capacities for ustiloxins A and B on resin SP700 were 83.76 mg and 29.77 mg, respectively at their breakthrough points.

#### 2.7.2. Dynamic Desorption Curves of Resins SP207and SP700 for Ustiloxins A and B

**Figure 4:**
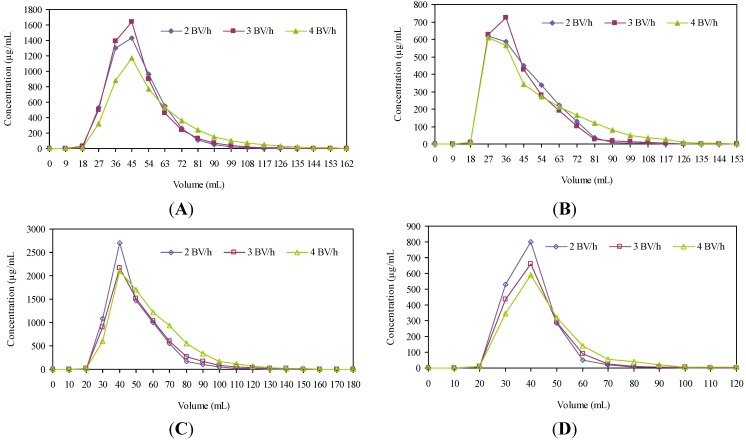
Dynamic desorption curves of ustiloxin A on the column packed with resin SP207 (**A**), ustiloxin B on the column packed with resin SP207 (**B**), ustiloxin A on the column packed with resin SP700 (**C**), and ustiloxin B on the column packed with resin SP700 (**D**).

The dynamic desorption curves of resins SP207 and SP700 for ustiloxins are shown in Figure 4. They were obtained based on the flow rates and volumes of desorption solution. 

The resin SP207 was eluted with 40% ethanol solution at a flow rate of 2, 3 and 4 BV/h, respectively. The results showed that the best desorption performance was obtained at a flow rate of 3 BV/h. At this flow rate, ustiloxins A and B could be eluted out at the same time by 4 BV (108 mL) of eluent (Figure 4A,B). Thus, 3 BV/h was selected as the proper desorption flow rate. Meanwhile, 4 BV was selected as the volumn of desorption solution in consideration of the lower volume consumption and high efficiency.

The resin SP700 was eluted with 30% ethanol solution at a flow rate of 2, 3 and 4 BV/h, respectively. The results showed that the best desorption performance was obtained at a flow rate of 2 BV/h. At this flow rate, ustiloxin A could be eluted out by 4 BV (120 mL) of eluent, and ustiloxin B could be eluted out by 3 BV (90 mL) of eluent (Figure 4C,D).

Based on the above results, the optimum separation process of ustiloxins A and B on resin SP207 was as follows: pH of the sample solution was 4; flow rate was 2 BV/h; processing volume was 32 BV; temperature was 25 °C. In the second step of desorption, ethanol concentration was 40%; pH of the desorption solution was 4; flow rate was 3 BV/h; eluent volume was 4 BV. The purities and recoveries of ustiloxins A and B in crude extract under optimum conditions by resin SP207 are shown in Table 6.

On the other hand, the optimum separation process of ustiloxins A and B on resin SP700 was as follows: pH of the sample solution was 4; flow rate was 2 BV/h; processing volume was 26 BV; temperature was 25 °C. In the second step of desorption, ethanol concentration was 30%; pH of desorption solution was 4; flow rate was 2 BV/h; eluent volume was 4 BV. The purities and recoveries of ustiloxins A and B in crude extract under optimum conditions by resin SP700 are shown in Table 6.

**Table 6:** Purities and recoveries of ustiloxins A and B separated with resins SP207 and SP700.

Adsorbate	Resin SP207		Resin SP700	
	Purity in the concentrate treated with resin (%)	Recovery (%)	Purity in the concentrate treated with resin (%)	Recovery (%)
Ustiloxin A	3.85	96.67	2.52	93.65
Ustiloxin B	1.87	81.25	1.47	88.64

Note: The purities of ustiloxins A and B in water extract of rice false smut were 0.16% and 0.09%, respectively.

**Figure 5:**
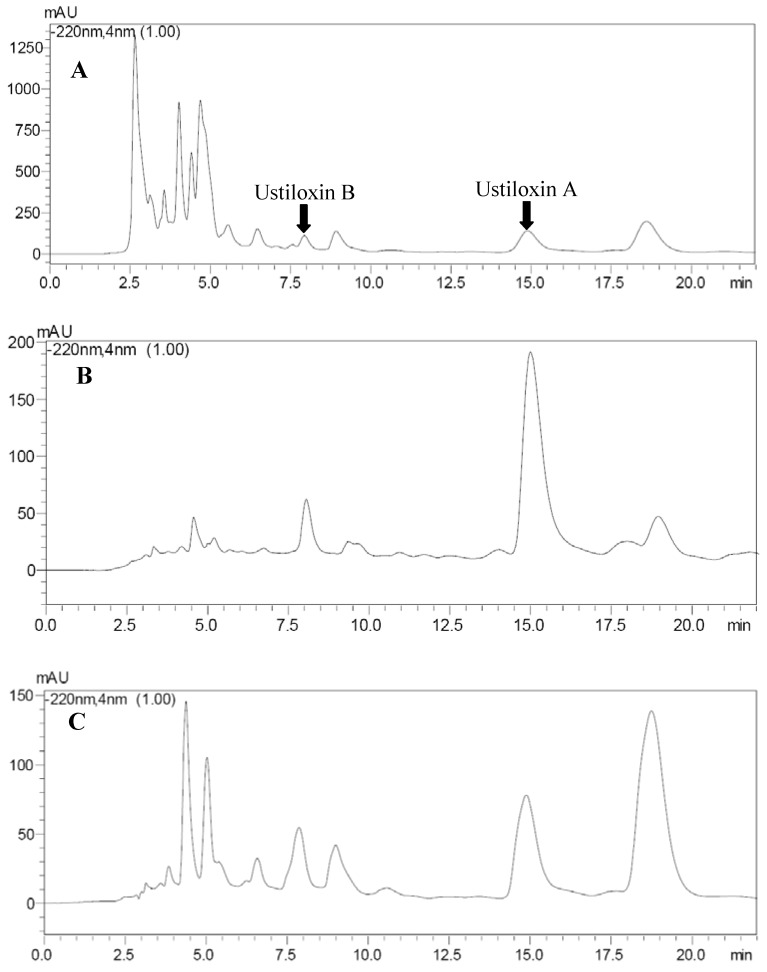
HPLC profiles of the samples untreated (**A**) and treated with resins SP207 (**B**) and SP700 (**C**). Retention time of ustiloxins A and B were 7.90 min and 14.95 min, respectively.

As can be seen from Table 6, after treatment with resin SP207, the purities of ustiloxins A and B increased to 3.85% and 1.87% from 0.16% and 0.09%, which corresponds to a 23.06-fold and 19.78-fold increase, respectively. Recovery rates were 96.67% for ustiloxin A and 81.25% for ustiloxin B. Similarly, after treatment with resin SP700, the purities of ustiloxins A and B raised to 2.52% and 1.47% from 0.16% and 0.09%, which were increased 14.75-fold and 15.33-fold, respectively. Recovery rates were 93.65% for ustiloxin A and 88.64% for ustiloxin B. The HPLC profiles of the sample solutions treated or untreated with resins SP207 or SP700 are illustrated in Figure 5. The purities of ustiloxins A and B were obviously increased.

## 3. Experimental

### 3.1. Materials, Chemicals and Reagents

Rice false smut balls were collected from the southwest of Shandong Province in China in October 2011, which were mainly composed of chlamydospores and mycelia of rice false smut pathogen (*Villosiclava virens*). The materials were left to dry in shade at room temperature to a constant weight, and were then stored in the sealed plastic bags at −20 °C until required.

The standards of ustiloxins A and B were isolated from rice false smut balls in our previous study [5]. For standard solution, two ustiloxins were dissolved in methanol-water (15:85, v/v) to yield the solution at 1.00 mg/mL, respectively, which were diluted with methanol-water (15:85, v/v) to obtain a series of appropriate concentrations for establishment of the calibration curves. Water was purified by a Mill-Q system (TTL-30C, Tongtai, Beijing, China). Methanol was at HPLC grade, and all the other reagents were at analytical grade. All solvents prepared for HPLC were filtered through a 0.45 µm nylon membrane and degassed with ultrasonication before use.

### 3.2. Macroporous Resins

One part of macroporous resins including X-5, D-101, HPD-100, D1300, D3520, AB-8, DM130, DS-8, DM-301, ADS-17, S-8, NKA-9 and DA-201 were purchased from Haiguang Chemical Industrial Company (Tianjin, China). Another part of resins including HP-20, SP825L, SP850, SP700, SP70, SP207 and HP_2_MGL were purchased from Mitsubishi Chemical Holdings (Tokyo, Japan). Their physical properties provided by the manufacturers are summarized in Table 7.

**Table 7:** Physical properties of the macroporous resins employed.

Macroporous resin	Polarity	Particle size (mm)	Surface area (m^2^/g)	Average pore diameter (nm)	Moisture content (%)
X-5	Non-polar	0.30–1.25	500–600	29–30	50.13 ± 0.17
D-101	Non-polar	0.30–1.25	480–530	9–11	67.89 ± 0.27
HPD-100	Non-polar	0.30–1.25	≥650	8–9	68.39 ± 0.48
D1300	Non-polar	0.30–1.25	≥600	9–10	68.27 ± 0.57
D3520	Non-polar	0.30–1.25	480–520	8–9	71.56 ± 0.18
AB-8	Weak-polar	0.30–1.25	480–520	13–14	68.33 ± 0.61
DM130	Weak-polar	0.30–1.25	500–550	9–10	66.00 ± 0.32
DS-8	Weak-polar	0.30–1.25	500–550	12–14	63.55 ± 0.10
DM-301	Middle-polar	0.30–1.25	≥330	14–17	65.48 ± 1.32
ADS-17	Middle-polar	0.30–1.25	90–150	25–30	55.16 ± 1.09
S-8	Polar	0.30–1.25	100–200	28–30	67.12 ± 0.35
NKA-9	Polar	0.30–1.25	250–290	15–16	70.22 ± 0.23
DA-201	Polar	0.30–1.25	≥200	10–13	70.64 ± 0.52
HP-20	Non-polar	≥ 0.25	590	38	60.25 ± 0.53
SP825L	Non-polar	≥ 0.25	930	14	57.63 ± 0.03
SP850	Non-polar	≥ 0.25	930	9	48.34 ± 0.05
SP700	Non-polar	≥ 0.25	1200	18	67.47 ± 0.05
SP70	Non-polar	≥ 0.25	870	14	61.24 ± 0.15
SP207	Non-polar	≥ 0.25	600	22	50.27 ± 0.49
HP_2_MGL	Middle-polar	≥ 0.30	570	48	64.87± 0.94

Note: Informations were provided by the manufactures except moisture content.

The resins were soaked in ethanol for 24 h and then washed with deionized water until there was no residue of ethanol. The moisture content of each resin was determined as follows. Three samples of each resin were weighted, and placed in a drying oven at 80 °C to constant dry weight. The moisture contents were then determined.

### 3.3. Preparaton of Crude Ustiloxin Extracts

The powdered sample (2 kg) of rice false smut balls was extracted three times with deionized water (6 L each time) under ultrasound for 30 min at room temperature. The water solution was concentrated under vacuum at 60 °C by a rotary evaporator to afford a concentrated water extract. The purities (contents) of ustiloxins A and B in the extract were determined as 0.16% and 0.09%, respectively by HPLC. The dry extract was stored at 4 °C until required.

### 3.4. HPLC Analysis of Ustiloxins A and B

Quantitative analysis of ustiloxins A and B by HPLC was carried out as described previously [5]. Ustiloxin purity (content) was analyzed by a Prominence LC-20A high-performance liquid chromatography (HPLC) system (Shimadzu, Japan), which consisted of two LC-20AT solvent delivery units, an SIL-20A autosampler, an SPD-M20A photodiode array detector, and a CBM-20Alite system controller. Chromatographic separations were performed at 30 °C using Synergi reversed-phase Hydro-C_18_ column (250 mm × 4.6 mm, 5 μm, Phenomenex, Torrance, CA, USA). The mobile phase, composed of methanol-water containing 0.02% trifluoacetic acid (TFA) (15:85, v/v), was eluted at a flow rate of 1.0 mL/min, with UV detection at 220 nm and a total analysis time of 20 min. The injection volume was 20 µL. The LC solution multi-PDA workstation was employed to acquire and process chromatographic data. The calibration curves of the two targeted ustiloxins, that showed good linearity, were *Y* = 2969445.7810*X* − 55531.7034 (*R* = 0.9998) (ustiloxin A) and *Y* = 2394672.3039*X* − 79066.0951 (*R* = 0.9998) (ustiloxin B), respectively, where *Y* is the peak area of analyte, and *X* is the injection quantity (μg) of analyte.

### 3.5. Static Adsorption and Desorption Tests

#### 3.5.1. Adsorption and Desorption Properties of Resins

The static adsorption process of the water extract from rice false smut balls on macroporous resins was carried out as follows: 1.0 g of resin (dry weight basis) was added to the conical flask containing 30 mL aqueous sample solution with the known concentrations of ustiloxins A and B. The flasks were sealed with parafilm and then shaken for 8 h at 100 rpm and 25 °C in a rotary shaker. The initial ustiloxins A and B’s concentrations of the sample solutions as well as their concentrations after adsorption were analyzed by HPLC.

The static desorption tests were performed as follows: After reaching adsorption equilibrium, the residual solution was removed, and then the adsorbate-laden resin was washed with 100 mL deionized water, shaken for 2 h at 100 rpm and 25 °C. At last, they were desorbed in 100 mL 95% ethanol solution. The flasks were shaken for 2 h at 100 rpm and 25 °C. The suitable resin was selected based on its adsorption capacity, adsorption ratio and desorption ratio. The equations for quantification of these parameters were expressed as follows:

Adsorption capacity:

*Q*_e_ = (*C*_0_ – *C*_e_) × (*V*_i_/W) (1)

Absorption ratio:

*A* (%) = (*C*_0_ – *C*_e_)/*C*_0_ × 100 (2)

Desorption ratio:

*D* (%) = *C*_d_ × [*V*_d_/ (*C*_0_ – *C*_e_) *V_i_*] × 100 (3)

where *Q*_e_ is the adsorption capacity at adsorption equilibrium (µg/g resin); *A* and *D* are the adsorption ratio (%) and desorption ratio (%), respectively; *C*_0_ and *C*_e_ are the initial and equilibrium concentrations of ustiloxins in the solutions, respectively (µg/mL); *C*_d_ is the concentration of the ustiloxins in the effluent solutions of desorption (µg/mL); *V*_i_ is the volume of the initial sample solution (mL); *V*_d_ is the volume of the desorption solution (mL); *W* is the weight of the dry resin (g).

#### 3.5.2. Static Adsorption Kinetics of the Selected Resins

The adsorption kinetics of the selected resins for ustiloxins was studied according to the static adsorption test described above, except the adsorption time was changed to 4 h until adsorption equilibrium was reached. The initial concentrations of ustiloxins A and B in the static adsorption kinetics experiments were 69.79 µg/mL and 46.43 µg/mL, respectively. The concentrations of two targeted ustiloxins in the sample solutions were monitored by HPLC at predetermined time intervals until equilibrium.

#### 3.5.3. Adsorption Isotherms

Adsorption isotherm data give information about the affinity between solutes and adsorbents. Langmuir and the Freundlich isotherms are often used to reveal the linearity fitting and to describe how ustloxins interact with the resins. The adsorption isotherms of ustiloxins A and B on the optimum resins were performed as follows: 30 mL of sample solutions at different concentrations were put into conical flasks, which contained 0.5 g pre-weighed resins (dry weight basis), and then the conical flasks were shaken for 8 h at 25, 30 and 35 °C. The initial and equilibrium concentrations of ustiloxins A and B in the sample solutions were determined by HPLC. The equilibrium adsorption isotherms on the resin were obtained, and their degrees of fitness to Langmuir equation and Freundlich equation were evaluated. Langmuir equation and Freundlich equation for describing the interaction of ustloxins with the resins were as follows [12]:

Langmuir isotherm equation:

*C*_e_/*Q*_e_ = *C*_e_/*Q*_max_ + 1/(*k*·*Q*_max_) (4)

Equation (4) can be rearranged to the following linear form:

1/*Q*_e_ = 1/(*K*_L_·*C*_e_) + 1/*Q*_max_ (5)

where *Q*_max_ is the theoretically calculated maximum adsorption capacity (µg/g resin); *k* and *K*_L_ are constants; *Q*_e_ is the adsorption capacity at adsorption equilibrium (µg/g resin) and *C_e_* is the equilibrium concentration of solutes in the solutions.

Freundlich isotherm equation:

*Q*_e_ = *K*_F_·*C*_e_^1/*n*^ (6)

A linearized form of equation (6) can be written as:

Log *Q*_e_ = 1/*n* log *C*_e_ + log *K*_F_ (7)

where *K*_F_ is a constant, an indicator of adsorption capacity; 1/*n* is an empirical constant related to the magnitude of the adsorption driving force; *Q*_e_ and *C*_e_ are the same as those defined in Equations (4) and (5).

#### 3.5.4. Effects of pH Values of Sample Solution on the Adsorption Capacity

The effects of initial pH values of sample solution on the adsorption capacity were also studied using the same procedure described in Section 3.5.1. The initial pH values of sample solution were 3, 4, 5, 6, 7, 8, 9 and 10, and the adsorption ratio was calculated to reveal the adsorption capacity.

#### 3.5.5. Effects of pH Values of Ethanol Solution on Desorption Ratios of Resins and Purities of Ustiloxins

The effects of pH values of ethanol solution on desorption ratios and purities of ustiloxins A and B were also studied using the same procedure described in Section 3.5.1. The pH values of ethanol solution were 3, 4, 5, 6, 7, 8, 9, 10 and 11. The effluent solutions were concentrated under vacuum at 60 °C. The purities (or concentrations) of ustiloxins A and B in desorption solutions were determined by HPLC.

#### 3.5.6. Effects of Ethanol Concentrations on Desorption Ratios of Resins and Purities of Ustiloxins

The effects of concentrations of ethanol solution on desorption ratios and purities of ustiloxins A and B were also studied using the same procedure described in Section 3.5.1. The concentrations of ethanol solution were 10%, 20%, 30%, 40%, 50% and 70%, respectively. The effluent solutions were concentrated under vacuum at 60 °C. The purities (or concentrations) of ustiloxins A and B in the desorption solution were determined by HPLC.

### 3.6. Dynamic Adsorption and Desorption of Ustiloxins A and B on Resins SP207 and SP700

Dynamic adsorption and desorption experiments were carried out in a glass column (16 mm × 400 mm) wet-packed with 20 g of pretreated resin (SP207 or SP700). The bed volume (BV) and length of the packed resin were 26 mL and 13 cm for SP207, 30 mL and 15 cm for SP700, respectively. In the dynamic adsorption test, the sample solution was loaded continuously on the glass column and then the test was performed at 2, 3 and 4 BV/h, respectively. The purities (or concentrations) of two targeted ustiloxins were determined by HPLC for analyzing the effluent fractions.

Once adsorption reached saturation, the loading of the sample solution was stopped. The columns were firstly washed with deionized water and then eluented with ethanol-water solution at the same flow rate. The purities (or concentrations) of the two targeted ustiloxins in the effluent solution were monitored by HPLC analysis. The effluent solutions were concentrated under vacuum at 60 °C. The recoveries and the purities of ustiloxins A and B in the concentrate treated with resin were calculated.

### 3.7. Statistical Analysis

All experiments were carried out in triplicate, and the results were represented by their mean values and the standard deviations (SD). The data were submitted to analysis of variance (one-way ANOVA) to detect significant differences by PROC ANOVA of SAS version 8.2. The term significant has been used to denote the differences for which *p* ≤ 0.05.

## 4. Conclusions

In this work, purification of ustiloxins A and B from a water extract of rice false smut balls was achieved by using macroporous resins. Both resins SP207 and SP700 were screened to show the best adsorption and desorption properties for ustiloxins A and B among 20 commercial macroporous resins. Dynamic adsorption and desorption tests were carried out to optimize the process parameters. The optimal conditions for SP207 adsorption were processing volume of 32 BV, pH value of 4, and flow rate of 2 BV/h. Those for SP207 desorption were ratio of ethanol to water of 40:60 (v/v), pH value of 4, eluent volume of 4 BV, and flow rate of 3 BV/h. The optimal conditions for SP700 adsorption were processing volume of 26 BV, pH value of 4, and flow rate of 2 BV/h. Those for SP700 desorption were ratio of ethanol to water solution of 30:70 (v/v), eluent volume of 4 BV, pH value of 4, and flow rate of 2 BV/h. Under the optimal conditions, the purities of ustiloxins A and B dealt with SP207 increased 23.06-fold and 19.78-fold, and their recoveries were 96.67% and 81.25%, respectively. Similarly, the purities of ustiloxins A and B dealt with SP700 increased 14.75-fold and 15.33-fold, and their recoveries were 93.65% and 88.64%, respectively. The results show that adsorption and desorption of resins SP207 and SP700 are effective strategies for purifying ustiloxins A and B from rice false smut balls. This is the first time that macroporous resins were systematically investigated for the simultaneous separation and purification of ustiloxins A and B. The method will provide a basis for large-scale preparation of ustiloxins A and B from rice false smut balls in order to meet the needs of their applications in agriculture and medicine industry. In addition, the adsorption and desorption mechanisms of the resins on ustiloxins needs to be studied in detail.
